# Leaving the parental home, cohabitation, and marriage after a hematologic malignancy in childhood—A register‐based cohort study from the SALiCCS research program

**DOI:** 10.1002/cam4.70067

**Published:** 2024-08-01

**Authors:** Liisa Korhonen, Hanna Mogensen, Friederike Erdmann, Maria Feychting, Line Elmerdahl Frederiksen, Elli Hirvonen, Anja Krøyer, Anniina Kyrönlahti, Nea Malila, Camilla Pedersen, Janne Pitkäniemi, Mats Talbäck, Mervi Taskinen, Jeanette Falck Winther, Laura Madanat‐Harjuoja

**Affiliations:** ^1^ Finnish Cancer Registry Helsinki Finland; ^2^ New Children's Hospital University of Helsinki and Helsinki University Hospital Helsinki Finland; ^3^ Unit of Epidemiology Institute of Environmental Medicine, Karolinska Institutet Stockholm Sweden; ^4^ Childhood Cancer Research Group Danish Cancer Institute Copenhagen Denmark; ^5^ Division of Childhood Cancer Epidemiology Institute of Medical Biostatistics, Epidemiology and Informatics, University Medical Center of the Johannes Gutenberg University Mainz Mainz Germany; ^6^ Department of Public Health University of Helsinki Helsinki Finland; ^7^ School of Health Sciences University of Tampere Tampere Finland; ^8^ Division of Pediatric Hematology, Oncology and Stem Cell Transplantation Helsinki University Hospital and University of Helsinki Helsinki Finland; ^9^ Department of Clinical Medicine, Faculty of Health Aarhus University and University Hospital Aarhus Denmark

**Keywords:** childhood cancer, cohabitation, leaving home, marriage, Nordic, population‐based cohort study, social outcomes

## Abstract

**Introduction:**

Transitioning to adulthood often involves achieving independence from the parental home. We assessed whether the likelihood of leaving the parental home, cohabitation, and marriage was similar between patients who experienced a hematologic malignancy at a young age and their peers.

**Methods:**

We identified 11,575 patients diagnosed with a hematologic malignancy under the age of 20 years between 1971 and 2011 in Denmark, Finland, and Sweden, 57,727 country‐, age‐, and sex‐matched population comparisons and 11,803 sibling comparisons and obtained annual information on family and marital status by linking to the statistical institute databases. Hazard ratios (HR) for leaving the parental home, cohabitation and marriage were estimated using Cox proportional hazards modeling.

**Results:**

Young adults with a history of a hematologic malignancy were slightly less likely to leave the parental home (HR 0.89; 95% confidence interval [CI] 0.86–0.92; HR 0.87 [95% CI 0.82–0.92]), cohabit with a nonmarital partner (HR 0.83 [95%CI 0.78–0.87]; HR 0.84 [95% CI 0.77–0.92]) and be married (HR 0.87 [95% CI 0.82–0.91]; HR 0.86 [95% CI 0.79–0.93]), compared with population comparisons and siblings, respectively.

**Conclusions:**

Our findings provide reassurance that young adults with a history of a hematologic malignancy show only a slight decrease in their likelihood of gaining independence from their childhood family and forming close interpersonal relationships compared to peers. While most patients are coping well in the long term, integrating structured psychosocial support into long‐term follow‐up is recommended to facilitate a timely and adequate transition into adulthood.

## 1 INTRODUCTION

Annually, nearly 300 children below the age of 20 years receive a diagnosis of hematologic malignancy in Denmark, Finland, and Sweden combined.^1^ Hematologic malignancies represent the most frequently diagnosed type of cancer in children, comprising over 40% of childhood cancers and are divided into two groups: leukemias and lymphomas. Incidence rates vary considerably by age.^2^ In the past few decades, over 90% of children have survived for at least 5 years after the diagnosis of most hematologic malignancies in the Nordic and most other high‐income countries.^3^, ^4^, ^5^

Albeit high survival rates, over 60% of individuals who survived a childhood hematologic malignancy experience chronic health conditions.^6^ In addition to somatic late effects, there is a heightened awareness regarding the psychosocial impact of childhood cancer. Individuals who have experienced childhood malignancies are found to be at a higher risk for psychiatric symptoms, such as anxiety, depression, suicidal ideation, or delayed psychosexual development, in comparison to controls. Additionally, previous research suggests that survivors with compromised physical health frequently encounter difficulties in mental well‐being and experience adverse social outcomes.^7^, ^8^, ^9^

An approach to assessing the long‐term psychosocial impacts of cancer involves evaluating social accomplishments or the achievement of life goals. Forming close interpersonal relationships and gaining independence from parents are important social milestones that represent an adjustment to adult life. It has been shown that cancer as a life‐threatening disease and related treatments may impact childhood cancer patients' ability to move out of their parental homes and establish relationships.^10^, ^11^, ^12^ However, previous research was limited by diagnostically heterogeneous or small samples, cross‐sectional designs, or lack of appropriate comparison groups.

In the Nordic countries, all patients with hematologic malignancies are treated with standardized protocols in public hospitals. As the registration of cancer cases is virtually complete, this group of patients forms a coherent population large enough to assess social outcomes. Information on these outcomes is available in the Nordic national population registers and linkage across various registers is possible as all citizens have unique personal identification codes.

We analyzed population‐based register data from Denmark, Finland, and Sweden, including 11,575 childhood and adolescent patients with hematologic malignancies. The aim was to investigate whether the social independence patterns of patients differ from those of their siblings and population‐based comparisons. We specifically focused on outcomes related to leaving the parental home, cohabitation, and marriage, as these factors contribute to independence and quality of life.

## 2 MATERIALS AND METHODS

### 2.1 Design and study population

The present study is part of the SALiCCS (Socioeconomic Consequences in Adult Life after Childhood Cancer in Scandinavia; www.saliccs.org) research program. SALiCCS is a register‐ and population‐based cohort study conducted in Denmark, Finland, and Sweden, aiming to explore the socioeconomic impacts and family characteristics among childhood cancer survivors.^13^

The Nordic countries maintain civil registration systems,^14^, ^15^, ^16^ complemented by various national population‐based administrative and health data registers providing individual‐level information.^17^ Health care systems in these countries are primarily publicly funded, allowing everybody access to high‐quality health services.^17^ A unique personal identification code given to each resident enables accurate individual‐level data linkage across registers.

**FIGURE 1:**
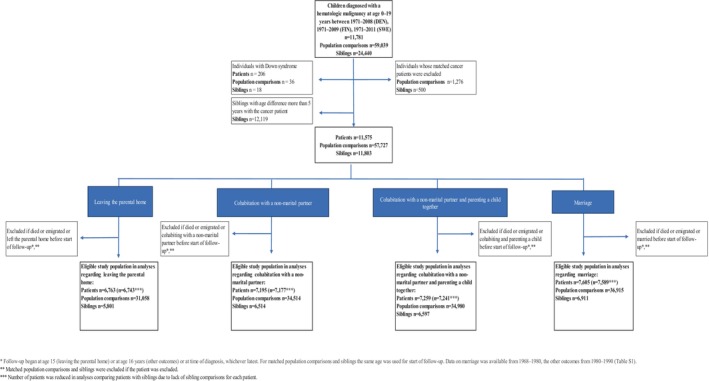
Flow chart.

Our study population included all patients diagnosed with a hematologic malignancy before the age of 20 years in Denmark 1971–2008, Finland 1971–2009, and Sweden 1971–2011. (Figure 1) Information on the date of diagnosis and cancer type was retrieved from the national cancer registers. Nordic cancer registers include population‐based data on every cancer diagnosis since 1943, 1953, and 1958 in Denmark, Finland, and Sweden, respectively. Mandatory reporting of new cancer cases ensures excellent coverage of these national registers.^18^, ^19^

Diagnoses of hematologic malignancies were classified into five categories according to the International Classification of Childhood Cancer (ICCC‐3)^20^: (1) Lymphoid leukemias, (2) Acute myeloid leukemias, (3) Other leukemias, myeloproliferative diseases, and myelodysplastic diseases, (4) Hodgkin's lymphoma, and (5) nonHodgkin's lymphoma and other lymphomas.^20^ Practically all cases of lymphoid leukemia in children are acute lymphoblastic leukemia (ALL). Due to differences in the registration of lymphomas between countries, nonHodgkin's lymphoma and other lymphomas were combined into one group.

Statistical institutes collect annual information on an individual's family status (e.g., spouse with or without children, cohabiting partner, child, etc.). Information on family status and marital status at the end of each year was obtained from national registers.^21^, ^22^, ^23^ Data availability is shown in Table S1. Information on the highest attained parental education was obtained from the statistical institutes of Denmark, Finland, and Sweden.^21^, ^22^, ^23^

We compared the cohort of children diagnosed with a hematologic malignancy with randomly selected population comparisons.^14^, ^21^, ^24^ Each comparison subject was matched to a respective patient based on birth year, sex, and country, maintaining a 1:5 ratio. (Figure 1).

For a second comparison group, we identified all siblings (full, half, and adoptive) of patients from the population registers,^14^, ^21^, ^24^ with a maximum allowable age difference of 5 years. Both siblings and population comparisons were required to have remained free of cancer up to the age of 20 years. Individuals with Down syndrome were excluded due to factors affecting the achievement of social outcomes, such as predisposition to cancer and intellectual disability. (Figure 1).

### 2.2 Social outcomes

The time point of leaving the parental home was defined as the first year during follow‐up when the person was no longer registered as a child in the family.

Cohabitation was examined on two separate levels: (1) Cohabiting with a nonmarital partner with or without children, and (2) cohabiting with a nonmarital partner and parenting a common child. The statistical offices defined cohabitation in two ways in Denmark and Finland: (1) Households with two individuals (over the age of 18 years in Finland) of the opposite sex, who were neither married, nor siblings, nor from the same family and had an age difference of less than 16 years in Finland and less than 15 years in Denmark, (2) Households with two individuals who were not married and parenting at least one common child, independent of their age difference. In Sweden, cohabitation was defined only using the latter definition.^21^, ^22^, ^23^ Cohabiting relationships of the same sex were excluded based on the definition of cohabitation established by statistical institutes.

We categorized marital status as follows: single, married, divorced, and widowed. Registered partnerships and gender‐neutral marriages were grouped under the married category. The category “divorced” was defined by the official dissolution of the marriage. Information on the highest parental education, classified based on the International Standard Classification of Education (ISCED),^25^ was documented for the year prior to or closest to the child's cancer diagnosis or the corresponding date for comparisons.

### 2.3 Statistical analysis

Depending on the outcome, follow‐up began on the date of cancer diagnosis for patients and population comparisons or at the start of availability of the respective data or at the age of 15 or 16 years, whichever occurred last. (Table S1) Follow‐up for siblings began on the date they were of the same age as the respective patient when diagnosed with a hematologic malignancy. For the analyses of leaving the parental home, follow‐up began at 15 years of age and for other outcomes at 16 years of age. All study subjects who had died, emigrated, or had attained the outcome of interest before the start of follow‐up were excluded from the analyses, as were their matched comparisons. Follow‐up ended at the specified outcome, death, emigration, or the end date of the study (August 11, 2017, in Denmark; December 31, 2014, in Finland; December 31, 2016 in Sweden), whichever occurred first. (Table S1) A flow chart indicating exclusion criteria is shown in Figure 1.

We estimated hazard ratios (HR) for the outcomes of interest with 95% confidence intervals (CIs) using Cox proportional hazards modeling with attained age as the underlying time scale. Cox regression models were adjusted for sex, country, and time period of follow‐up. When comparing cancer patients with population comparisons, the Cox models were further adjusted for the highest attained parental education. In analyses comparing cancer patients with siblings, the baseline hazard was stratified by the matched sibling pairs. Outcome data were analyzed separately for the initial occurrences of leaving the parental home, cohabitation, and marriage.

Covariates used in stratified analyses included cancer type, decade of cancer diagnosis, age at cancer diagnosis, sex, country, and highest parental education. We divided the patients' cancer diagnosis timeline into four decades: 1971–1979, 1980–1989, 1990–1999 and 2000–2011. The age at cancer diagnosis was classified into four categories: 0–4, 5–9, 10–14 and 15–19 years.

Additionally, we performed a sub‐analysis specifically focusing on patients with lymphoid leukemia. Treatment protocols have evolved over time, with central nervous system (CNS) irradiation gradually being omitted after 1992. In this sub‐analysis, patients were divided into two groups based on the treatment period: (1) those treated before 1992 and (2) those treated after 1992.^26^

Statistical analyses were conducted using the R statistical software, version 4.1.0.

## 3 RESULTS

Our study population comprised 11,575 children with a hematologic malignancy, 57,727 population comparisons and 11,803 sibling comparisons. (Figure 1) Among patients and comparisons, 58% were male and 42% female. (Table 1) The most common diagnoses were lymphoid leukemia (48.9%), Hodgkin lymphoma (17.1%) and acute myeloid leukemia (10.1%) (Table 1), reflecting the pattern and distribution of hematologic malignancies in this age‐ group.^2^

**TABLE 1:** Basic characteristics of patients with a hematologic malignancy, population comparisons and sibling comparisons.

	Patients		Population comparisons		Sibling comparisons	
	*n* = 11,575		*n* = 57,727		*n* = 11,803	
	*n*	%	*n*	%	*n*	%
Total	11,575	100	57,727	100	11,803	100
Country						
Denmark	3017	26·1	15,020	26.0	3025	25.6
Finland	3326	28·7	16,620	28.8	3479	29.5
Sweden	5232	45·2	26,087	45.2	5299	44.9
Sex						
Male	6709	58·0	33,457	58.0	5990	50.7
Female	4866	42·0	24,270	42.0	5813	49.3
Cancer diagnostic groups, according to ICCC3^a^						
Lymphoid leukemias	5658	48·9	28,208	48.9	5765	48.8
Acute myeloid leukemia	1166	10·1	5815	10.1	1310	11.1
Other leukemias	791	6·8	3947	6.8	787	6.7
Hodgkin lymphoma	1979	17·1	9871	17.1	1942	16.5
NonHodgkin and other lymphomas	1981	17.1	9886	17.1	1999	16.9
Period of diagnosis or equivalent date for siblings and matched individuals						
1971–1979	2481	21·4	12,336	21.4	2636	22.3
1980–1989	2735	23·6	13,658	23.7	2690	22.8
1990–1999	3038	26·2	15,106	26.2	3029	25.7
2000–2011^b^	3321	28.7	16,627	28.8	3448	29.2
Age at diagnosis for patients or equivalent date for siblings and matched individuals, years						
<1	419	3·6	2093	3.6	550	4.7
1–4	3465	29.9	17,245	29.9	3558	30.1
5–9	2409	20·8	12,014	20.8	2368	20.1
10–14	2179	18·8	10,877	18.8	2192	18.6
15–19	3103	26·8	15,498	26.8	3135	26.6

^a^ International Classification of Childhood Cancer, 3rd edition.

^b^ Denmark: 2008, Finland: 2009, Sweden: 2011.

### 3.1 Leaving home

**FIGURE 2:**
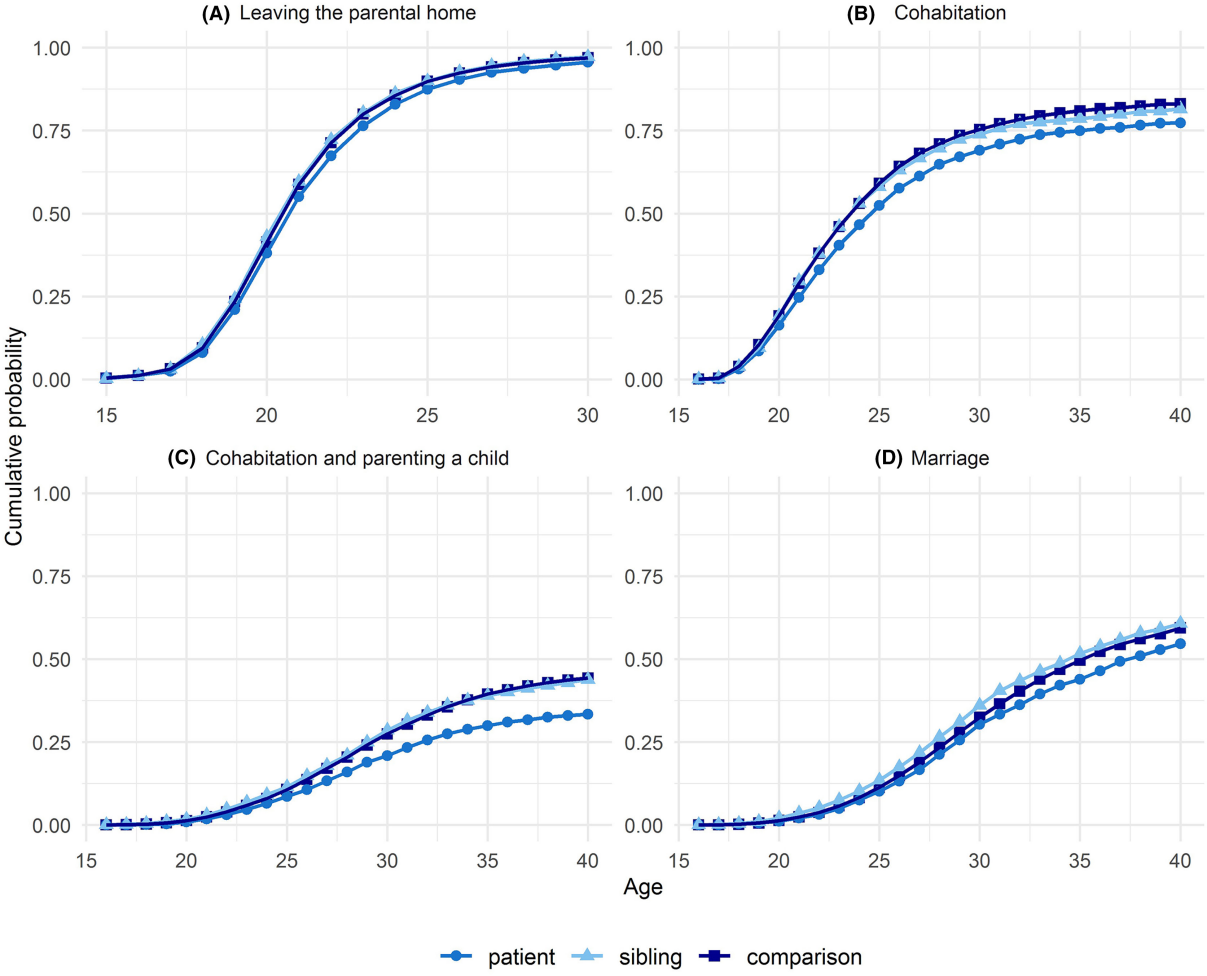
Cumulative probability of social outcomes by age at first outcome.

The age at which 50% of individuals had left the parental home was 20 years among patients, population comparisons and siblings. By age 30 years, the cumulative probability of leaving the parental home was over 90% in all three groups. (Figure 2).

Overall, children with a hematologic malignancy were 11% and 13% less likely to leave the parental home compared with population comparisons and siblings, respectively (HR 0.89, 95% CI 0.86–0.92; HR 0.87, 95% CI 0.82–0.92, Table 2A). In analyses by decade of cancer diagnosis, the effect estimates for leaving the parental home were less pronounced in later decades comparing cancer patients with population comparisons (1970s: HR 0.76, 95% CI 0.67–0.85; 1980s: HR 0.84, 95% CI 0.79–0.90; 1990s: HR 0.92, 95% CI 0.88–0.97; 2000s: HR 0.92, 95% CI 0.86–0.98). The analyses for population comparisons and siblings yielded similar results. (Table 2A).

**TABLE 2A:** Numbers and hazards ratios (HR) for outcomes leaving the parental home and marriage with 95% confidence intervals (CI) comparing patients with population comparisons and sibling comparisons.

	Leaving the parental home		Marriage	
	Patients vs. population comparisons	Patients vs. siblings	Patients vs. population comparisons	Patients vs. siblings
	Patients *n* = 6763, population comparisons *n* = 31,058	Patients *n* = 6743, siblings *n* = 5801	Patients *n* = 7605, population comparisons *n* = 36,915	Patients *n* = 7589, siblings *n* = 6911
	HR (95% CI)^b^	HR (95% CI)^c^	HR (95% CI)^b^	HR (95% CI)^c^
Total	0·89 (0·86–0·92)	0·87 (0·82–0·92)	0·87 (0·82–0·91)	0·86 (0·79–0·93)
5‐year survivors	0·89 (0·86–0·92)	0·85 (0·80–0·91)	0·87 (0·83–0·92)	0·86 (0·79–0·93)
Country				
Denmark	0·89 (0·84–0·94)	0·86 (0·76–0·96)	0·88 (0·80–0·97)	0·78 (0·66–0·92)
Finland	0·85 (0·80–0·91)	0·85 (0·76–0·95)	0·87 (0·79–0·96)	0·80 (0·69–0·93)
Sweden	0·90 (0·86–0·94)	0·88 (0·81–0·97)	0·86 (0·80–0·93)	0·96 (0·84–1·08)
Sex				
Male	0·89 (0·85–0·93)	0·87 (0·79–0·97)	0·90 (0·84–0·97)	0·88 (0·76–1·03)
Female	0·89 (0·85–0·93)	0·92 (0·81–1·04)	0·84 (0·78–0·90)	0·85 (0·72–1·00)
Cancer diagnostic groups, according to ICCC3^a^				
Leukemia	0·87 (0·83–0·91)	0·86 (0·80–0·93)	0·75 (0·70–0·81)	0·80 (0·71–0·90)
Lymphoid leukemias	0·88 (0·84–0·93)	0·86 (0·79–0·94)	0·74 (0·68–0·81)	0·77 (0·68–0·88)
Acute myeloid leukemia	0·85 (0·74–0·97)	0·88 (0·68–1·13)	0·79 (0·63–1·00)	1·20 (0·83–1·72)
Other leukemias	0·73 (0·61–0·88)	0·78 (0·55–1·12)	0·72 (0·54–0·98)	0·65 (0·40–1·04)
Lymphoma	0·91 (0·87–0·95)	0·88 (0·80–0·96)	0·98 (0·92–1·05)	0·91 (0·81–1·02)
Hodgkin lymphoma	0·91 (0·85–0·97)	0·89 (0·79–1·01)	1·01 (0·93–1·10)	0·84 (0·73–0·97)
NonHodgkin and other lymphomas	0·91 (0·84–0·98)	0·86 (0·74–0·99)	0·94 (0·84–1·05)	1·05 (0·87–1·28)
Period of cancer diagnosis				
1971–1979	0·76 (0·67–0·85)	0·71 (0·56–0·90)	0·76 (0·68–0·86)	0·77 (0·65–0·91)
1980–1989	0·84 (0·79–0·90)	0·87 (0·78–0·98)	0·81 (0·74–0·88)	0·82 (0·72–0·94)
1990–1999	0·92 (0·88–0·97)	0·89 (0·81–0·98)	1·01 (0·93–1·11)	0·91 (0·79–1·06)
2000–2011 (DEN: −2008, FIN: −2009, SWE: −2011)	0·92 (0·86–0·98)	0·87 (0·77–0·98)	0·95 (0·80–1·11)	1·21 (0·91–1·62)
Age at patient's cancer diagnosis, years				
0–4	0·89 (0·84–0·95)	0·85 (0·77–0·95)	0·68 (0·60–0·77)	0·73 (0·60–0·88)
5–9	0·88 (0·82–0·94)	0·82 (0·72–0·93)	0·74 (0·65–0·84)	0·67 (0·55–0·82)
10–14	0·87 (0·82–0·94)	0·94 (0·82–1·08)	0·91 (0·82–1·02)	1·07 (0·89–1·28)
15–19	0·90 (0·85–0·96)	0·88 (0·78–0·99)	1·00 (0·92–1·08)	0·92 (0·81–1·05)

^a^ International Classification of Childhood Cancer, 3rd edition.

^b^ Statistical models were adjusted for sex, country, time period for follow‐up, and highest parental education. Highest parental education was grouped as: (1) Early childhood education, primary education, and lower secondary education (ISCED levels 0–2), (2) General upper secondary education (ISCED 3), (3) Higher levels of education (ISCED 4–8).

^c^ Statistical models were adjusted for sex, country, and time period for follow‐up.

### 3.2 Cohabitation

The age at which 50% of individuals were cohabiting was 24 years among patients and 23 years among siblings and population comparisons. (Figure 2) Children with a hematologic malignancy were 17% and 16% less likely to cohabit with a nonmarital partner compared with population and sibling comparisons, respectively (HR 0.83, 95% CI 0.78–0.87; HR 0.84, 95% CI 0.77–0.92). The likelihood was, similarly, significantly decreased in analyses stratified by country, sex, period of cancer diagnosis, and age at cancer diagnosis. In analyses stratified by cancer type, we found a statistically significant decrease in the likelihood of cohabitation in patients diagnosed with lymphoid leukemia, Hodgkin lymphoma, and other lymphomas (HR 0.78, 95% CI 0.73–0.85; HR 0.87, 95% CI 0.79–0.96; HR 0.83, 95% CI 0.73–0.93). (Table 2B) The cumulative probability of cohabitation appeared lower among patients with a hematologic malignancy when compared with comparisons. (Figure 2).

**TABLE 2B:** Numbers and hazards ratios (HR) for outcome cohabitation with 95% confidence intervals (CI) comparing patients with population comparisons and sibling comparisons.

	Cohabitation with a nonmarital partner		Cohabitation with a nonmarital partner and parenting a child together	
	Patients vs. population comparisons	Patients vs. siblings	Patients vs. population comparisons	Patients vs. siblings
	Patients *n* = 7195, population comparisons *n* = 34,514	Patients *n* = 7177, siblings *n* = 6514	Patients *n* = 7259, population comparisons *n* = 34,980	Patients *n* = 7241, siblings *n* = 6597
	HR (95% CI)^b^	HR (95% CI)^c^	HR (95% CI)^b^	HR (95% CI)^c^
Total	0·83 (0·78–0·87)	0·84 (0·77–0·92)	0·72 (0·67–0·76)	0·66 (0·60–0·73)
5‐year survivors	0·82 (0·78–0·87)	0·82 (0·75–0·91)	0·72 (0·68–0·77)	0·68 (0·61–0·75)
Country				
Denmark	0·83 (0·78–0·89)	0·86 (0·76–0·98)	0·73 (0·65–0·83)	0·73 (0·59–0·89)
Finland	0·81 (0·75–0·87)	0·82 (0·73–0·93)	0·78 (0·68–0·90)	0·71 (0·58–0·88)
Sweden	—	—	0·68 (0·62–0·74)	0·62 (0·54–0·72)
Sex				
Male	0·82 (0·77–0·88)	0·84 (0·71–0·99)	0·67 (0·62–0·73)	0·60 (0·50–0·72)
Female	0·83 (0·77–0·90)	0·79 (0·66–0·94)	0·76 (0·70–0·83)	0·67 (0·54–0·81)
Cancer diagnostic groups, according to ICCC3^a^				
Leukemia	0·80 (0·75–0·86)	0·81 (0·72–0·91)	0·71 (0·65–0·77)	0·64 (0·56–0·74)
Lymphoid leukemias	0·78 (0·73–0·85)	0·78 (0·68–0·89)	0·74 (0·67–0·81)	0·66 (0·57–0·77)
Acute myeloid leukemia	0·82 (0·67–1·01)	0·97 (0·67–1·41)	0·55 (0·41–0·74)	0·51 (0·31–0·83)
Other leukemias	1·16 (0·85–1·59)	1·01 (0·53–1·93)	0·57 (0·39–0·84)	0·70 (0·37–1·33)
Lymphoma	0·86 (0·79–0·92)	0·86 (0·75–0·98)	0·72 (0·66–0·79)	0·70 (0·60–0·81)
Hodgkin lymphoma	0·87 (0·79–0·96)	0·88 (0·74–1·04)	0·67 (0·60–0·76)	0·63 (0·52–0·77)
NonHodgkin and other lymphomas	0·83 (0·73–0·93)	0·83 (0·67–1·03)	0·81 (0·71–0·93)	0·79 (0·63–0·99)
Period of cancer diagnosis				
1971–1979	0·65 (0·55–0·76)	0·80 (0·62–1·04)	0·57 (0·48–0·67)	0·59 (0·46–0·77)
1980–1989	0·79 (0·72–0·86)	0·71 (0·60–0·84)	0·72 (0·65–0·80)	0·65 (0·55–0·76)
1990–1999	0·92 (0·84–0·99)	0·95 (0·82–1·10)	0·78 (0·71–0·87)	0·75 (0·63–0·89)
2000–2011 (DEN: −2008, FIN: −2009, SWE: −2011)	0·81 (0·72–0·91)	0·88 (0·71–1·08)	0·71 (0·60–0·84)	0·68 (0·50–0·93)
Age at patient's cancer diagnosis, years				
0–4	0·78 (0·70–0·86)	0·76 (0·63–0·90)	0·75 (0·66–0·86)	0·65 (0·52–0·80)
5–9	0·81 (0·72–0·91)	0·75 (0·61–0·93)	0·77 (0·68–0·89)	0·72 (0·57–0·89)
10–14	0·82 (0·74–0·91)	0·91 (0·75–1·10)	0·70 (0·62–0·81)	0·62 (0·50–0·78)
15–19	0·88 (0·81–0·96)	0·92 (0·78–1·07)	0·67 (0·60–0·74)	0·67 (0·57–0·80)

^a^ International Classification of Childhood Cancer, 3rd edition.

^b^ Statistical models were adjusted for sex, country, time period for follow‐up, and highest parental education. Highest parental education was grouped as: (1) Early childhood education, primary education, and lower secondary education (ISCED levels 0–2), (2) General upper secondary education (ISCED 3), (3) Higher levels of education (ISCED 4–8).

^c^ Statistical models were adjusted for sex, country, and time period for follow‐up.

The likelihood of cohabitation with a nonmarital partner and parenting a common child was also decreased when comparing patients with population and sibling comparisons (HR 0.72, 95% CI 0.67–0.76; HR 0.66, 95% CI 0.60–0.73, Table 2B).

### 3.3 Marriage

The age at which 50% of individuals were married was 37 years for cancer patients and 35 years for population comparisons and siblings. (Figure 2) The median age reached by the end of follow‐up was 27 years for cancer patients and 26 years for both comparison groups. (Table S2).

Patients with a hematologic malignancy were 13% and 14% less likely to be married by the end of follow‐up when compared to population and sibling comparisons, respectively (HR 0.87, 95% CI 0.82–0.91; HR 0.86, 95% CI 0.79–0.93, Table 2A). In analyses stratified by cancer type, we found a statistically significantly decreased likelihood of marriage in leukemia patients only (HR 0.75, 95% CI 0.70–0.81) and in analyses stratified by age at cancer diagnosis, the likelihood of marriage was statistically significantly decreased in patients diagnosed at 0–4 and 5–9 years of age, when compared with population comparisons (HR 0.68, 95% CI 0.60–0.77; HR 0.74, 95% CI 0.65–0.84).

In stratified analyses comparing patients with sibling comparisons, a statistically significant decrease in the likelihood of getting married was observed in patients with lymphoid leukemia and Hodgkin's lymphoma (HR 0.77, 95% CI 0.68–0.88: HR 0.84, 95% CI 0.73–0.97), in patients diagnosed at 0–4 and 5–9 years of age (HR 0.73, 95% CI 0.60–0.88; HR 0.67, 95% CI 0.55–0.82), in patients diagnosed in the 1970s and 1980s (HR 0.77, 95% CI 0.65–0.91; HR 0.82, 95% CI 0.72–0.94), and in patients from Denmark and Finland (HR 0.78, 95% CI 0.66–0.92; HR 0.80, 95% CI 0.69–0.93). (Table 2A).

Restricting the cohort to five‐year survivors, the overall results regarding all measures of social independence remained similar. (Table 2A) In the sub‐analysis comprising patients with lymphoid leukemia and matched population comparisons, the likelihood for all social outcomes was statistically significantly decreased in patients treated before 1992. In patients treated after 1992, we observed a statistically significant difference only regarding cohabitation with a nonmarital partner when comparing patients with population comparisons. (Table S3).

## 4 DISCUSSION

In this population‐based cohort study comprising over 11,000 patients with a hematologic malignancy in childhood from Denmark, Finland, and Sweden, we observed a slightly reduced likelihood of leaving the parental home, cohabitation with a nonmarital partner, and marriage in patients compared with population and sibling comparisons. The median age at first attainment of these measures of social independence showed no notable difference between patients and either of the comparison groups in any of the studied outcomes.

Developing relationships outside of the family and gaining independence are age‐specific developmental milestones that signify the transition to adulthood. However, due to prolonged hospitalizations and isolation resulting from childhood cancer treatment, young individuals diagnosed with childhood cancer often face significant disruptions in their social lives. This disturbance during a critical and sensitive stage of life can affect the normal developmental trajectory of childhood and adolescence.^27^, ^28^ Additionally, childhood and adolescence are recognized as vulnerable periods for the onset of mental disorders.^29^

The distribution of hematologic malignancies differs considerably by age at diagnosis. In children diagnosed with a hematologic malignancy under the age of 5 years, nearly 90% of cases are leukemias, whereas in adolescents, 40% are leukemias, and the rest are lymphomas.^2^ Treatment of hematologic malignancies has been standardized in the Nordic countries since the early 1980s.^26^, ^30^ Prior to the advent of common Nordic treatment protocols in 1992, prophylactic cranial irradiation was a standard treatment for children diagnosed with ALL who faced a high risk of CNS relapse.^26^ In previous studies comprising the entire spectrum of childhood malignancies, patients with a tumor or treatment affecting the CNS have been at the highest risk regarding adverse social consequences.^11^, ^31^

In our study, patients treated in the 1970s showed a reduced likelihood of leaving home, cohabiting, or marrying relative to comparisons. However, this difference was less notable among those treated in the 1990s. Prophylactic CNS irradiation in ALL patients may have influenced earlier adverse psychosocial outcomes, but it could also suggest recent improvements in psychosocial care and family support.

When studying individuals' ability to gain independence from their parents, considering socio‐economic status is important. In this study of adolescents and young adults with a history of childhood malignancy, we utilized parental education as a proxy for family socio‐economic status. Using socioeconomic information directly from young cancer patients themselves is problematic, as many of the studied outcomes manifest before individuals reach their final education, income, or occupation stages. In our analyses, parental education did not impact the results. However, we still decided to present the results adjusted for highest parental education as they are more easily comparable with analyses comparing patients with siblings, where parental education is adjusted for by design.

As there were no children who had left home before the age of 15 years in our data from Finland and only a few in the Danish and Swedish data, we restricted the start of follow‐up to 15 years regarding this outcome. However, as we found marriages from the age of 16 years in our data, we restricted the age for possible marriage to start from 16 years in this study. These age points for the start of follow‐up were chosen to lose as little available data as possible. Due to our matched study design, these restrictions are not expected to introduce bias to our results.

### 4.1 Leaving the parental home

In Northern European nations, young individuals typically depart from their parental homes at a relatively young age, and there is minimal disparity between sexes. This is unlike other European countries where young women often leave the parental home earlier than men.^32^ In our study, the median age at leaving the parental home was 1 year younger in females than in males among patients and both comparison groups (data not shown).

A Childhood Cancer Survivor Study (CCSS) substudy in North America showed that survivors of childhood leukemia were less likely to live independently compared with siblings.^10^ Our findings are in line with these results. However, in contrast to our study, a Danish register study exploring childhood cancer patients diagnosed in 1965–1995 did not find a statistically significant difference in leaving the parental home when comparing survivors of hematologic malignancies with the matched control cohort.^31^ Even though the population of this Danish study^31^ also contributed to our cohort, the difference in results may be explained by our larger cohort of patients and longer follow‐up.

Although the risk for not leaving the parental home was statistically higher for individuals with a hematologic malignancy in childhood compared with both comparison groups, the cumulative probability of leaving the parental home by age 30 years was nearly 95% in all three groups. (Figure 2).

### 4.2 Cohabitation and marriage

While marriage rates have decreased considerably over the last decades, the average age at first marriage has increased in Europe. Concurrently, cohabitation and various forms of partnership arrangements are becoming more prevalent in all of the Nordic countries, and more couples become parents outside of wedlock.^32^

Few studies have explored these outcomes, specifically among survivors of childhood hematologic malignancies. Similar to our study, a questionnaire‐based study from the United Kingdom showed that children with ALL had fewer close relationships than the general population.^33^

Two North American questionnaire studies^34^, ^35^ found leukemia patients treated with cranial irradiation to have a lower likelihood of marriage compared with siblings^34^ and population controls.^34^, ^35^ A Swedish register study of childhood ALL patients also found a lower marital rate when compared with matched population controls.^36^ In line with the American studies, there was no notable difference between the survivors who were not irradiated and their controls. A recent CCSS publication showed childhood acute myeloid leukemia (AML) survivors to be at increased risk for remaining unmarried or not having a partner regardless of treatment type.^37^ In our study, we confirmed a lower likelihood of marriage for leukemia survivors, especially during later time periods, compared with population comparisons, although the results were not statistically significant among patients with AML. This association was not observed in patients with lymphoma. Information on treatment was not available in the register data.

### 4.3 Strengths and limitations

The major strength of this study is its use of extensive and distinctive data from population registers in three Nordic countries, ensuring virtually complete follow‐up and freedom from selection or participation biases. Its design facilitated reliable annual evaluation of social outcomes. Moreover, including two comparison groups allowed for controlling potential confounders arising from shared genetic and environmental factors, such as parental socioeconomic background. To ensure the comparability of patients and siblings in terms of the time periods they lived through, the age difference was limited to a maximum of 5 years. This restriction allows for the examination of trends in factors that evolve over time in society, such as marriage. Moreover, in previous studies, the study population has often been limited by diagnostic heterogeneity. Restricting the study population to the most common group of childhood cancers, hematologic malignancies, enabled us to focus on a homogenous group of patients.

In this study, the definition of outcomes varied slightly between the three countries, but as comparisons were made within matched sets, the overall results were not affected. Analyses regarding cohabitation were conducted with Danish and Finnish data only, which somewhat reduced statistical power. On the other hand, information on cohabitation with a nonmarital partner and parenting a child together was available from all three countries. The interpretation of these results should, however, be made with caution as these results also reflect fertility which is overall reduced among patients with a history of a hematologic malignancy.^36^, ^38^

In the Nordic registers, individuals, most commonly students, who have moved from their parental home may still have been registered at their parents' place of residence, which may be a source of classification bias in our study. On the other hand, children who were living at boarding schools or long‐term medical institutes may have been registered as living independently from their childhood families. It is unlikely that this would have affected the results significantly as we assume that in the majority of cases, registration indicates the true place of residence, and the same classification bias may have been present in the population and sibling comparisons.

When investigating social outcomes utilizing information at the end of each year, changes in status during the same calendar year will be missed. However, we postulate that these quick changes would be rather rare and thus would not have introduced significant bias to our results. Additionally, due to unavailability of the data, we cannot be sure if the first registered outcome event in our data is de facto the person's first event, which would affect mainly older individuals diagnosed in the earlier time periods. Many individuals in our cohort, especially during the most recent diagnostic periods, were so young that they did not have the chance to reach the social outcome, similarly among patients and comparisons.

## 5 CONCLUSIONS

In conclusion, our study's reassuring findings indicate that young adults with a history of childhood hematologic malignancies only exhibit a slightly decreased likelihood of independent living, nonmarital cohabitation, and marriage compared to their peers. While survival rates are excellent and most childhood hematologic malignancy patients cope well in the long term, it is crucial for young cancer patients to actively engage in typical adolescent and young adult activities to minimize the impact of childhood cancer and improve their health‐related quality of life. They should have easy access to psychosocial support to navigate the developmental challenges associated with this life stage. Creating a sense of normalcy is essential, and adaptable healthcare environments that recognize the significant role of peers are vital in achieving this goal. This approach will facilitate timely and adequate independence from childhood families for these young individuals.

## AUTHOR CONTRIBUTIONS

**Liisa Korhonen:** Conceptualization (equal); data curation (equal); formal analysis (equal); investigation (equal); methodology (equal); writing – original draft (lead). **Hanna Mogensen:** Conceptualization (equal); data curation (equal); writing – review and editing (equal). **Friederike Erdmann:** Conceptualization (equal); data curation (equal); writing – review and editing (equal). **Maria Feychting:** Conceptualization (equal); data curation (equal); funding acquisition (equal); project administration (equal); writing – review and editing (equal). **Line Elmerdahl Frederiksen:** Data curation (equal); writing – review and editing (equal). **Elli Hirvonen:** Data curation (equal); formal analysis (equal); methodology (equal); software (equal); writing – review and editing (equal). **Anja Krøyer:** Data curation (equal); software (equal); writing – review and editing (equal). **Anniina Kyrönlahti:** Data curation (equal); writing – review and editing (equal). **Nea Malila:** Conceptualization (equal); data curation (equal); funding acquisition (equal); methodology (equal); project administration (equal); supervision (equal); writing – review and editing (lead). **Camilla Pedersen:** Data curation (equal); writing – review and editing (equal). **Janne Pitkäniemi:** Data curation (equal); formal analysis (equal); methodology (equal); writing – review and editing (equal). **Mats Talbäck:** Data curation (equal); software (equal); writing – review and editing (equal). **Mervi Taskinen:** Conceptualization (equal); data curation (equal); methodology (equal); supervision (equal); writing – review and editing (lead). **Jeanette Falck Winther:** Conceptualization (equal); data curation (equal); funding acquisition (equal); project administration (lead); writing – review and editing (equal). **Laura Madanat‐Harjuoja:** Conceptualization (equal); data curation (equal); methodology (equal); supervision (equal); writing – review and editing (equal).

## CONFLICT OF INTEREST STATEMENT

The authors declare no conflict of interest.

## ETHICS STATEMENT

The SALiCCS research program has been approved by Statistics Denmark, the Regional Ethical Review Board in Stockholm, Sweden (dnr 2016/25–31/5, 2016/1561–32, 2017/1656–32, 2017/1990–32, 2017/2340–32, 2018/1165–32), Findata (Dnro THL/5543/14.06.00/2020) prolonging the former approvals by the National Institute for Health and Welfare and Social Insurance Institution (KELA), and Statistics Finland (extension of license TK/3501/07.03.00/2021, former TK‐53‐394‐17) in Finland. For the European Union General Data Protection Regulation (GDPR), the SALiCCS project is listed in a local archive (2018‐DCRC‐0044) at the Danish Cancer Society Research Center.

## Supporting information


Table S1.

## Data Availability

The data that support the findings of this manuscript were securely accessed through a dedicated remote platform at Statistics Denmark. After receiving the necessary ethical approvals (where applicable) and undergoing a secrecy assessment, we acquired pseudonymized individual‐level data from the respective national register authorities. In alignment with the regulations of Denmark, Finland and, Sweden, access to individual‐level sensitive data is restricted to researchers who fulfill the legal requirements for handling such confidential information. For any inquiries regarding data access, please contact Jeanette Falck Winther (jeanette@cancer.dk), the Principal Investigator of the SALiCCS research program.
