# Supplementary material for: Leaving the parental home, cohabitation, and marriage after a hematologic malignancy in childhood—A register‐based cohort study from the SALiCCS research program
Source: Cancer Med. 2024 Aug 1;13(15):e70067. doi: 10.1002/cam4.70067 (PMC11292853; doi:10.1002/cam4.70067)
Supplement: Supplementary file 1 — Table S1. [file CAM4-13-e70067-s001.pdf]

Leaving the parental home, cohabitation, and marriage after a hematologic malignancy in childhood – A register-based cohort study from the SALiCCS research program

Supplementary Material, Table of Contents

Table S1. Time periods of inclusion, follow-up, and availability of the outcome information in all countries. 2

Table S2. Characteristics of patients with a hematologic malignancy, population comparisons, and sibling comparisons. 3

Table S3. Hazards ratios (HR) for outcomes leaving the parental home, cohabitation, and marriage with 95 % confidence intervals (CI) comparing patients with lymphoid leukemia with population comparisons. 4

Table S1. Time periods of inclusion, follow-up, and availability of the outcome information in all countries

Country	Period of cancer diagnosis	End of the follow-up period	Availability of annual data regarding leaving the parental home, and cohabitation	Availability of annual data regarding marriages
Denmark	1971 – 2008	Aug 11th 2017	1980 – 2018 [a]	1980 – 2018 [a]
Finland	1971 – 2009	Dec 31st 2014	1987 – 2014 [b]	1970 – 2014 [c]
Sweden	1971 – 2011	Dec 31st 2016	1990 – 2016 [d]	1968 – 2016 [d]

Data source:

a) Statistics Denmark

b) Statistics Finland

c) The Finnish Digital and Population Data Services Agency

d) Statistics Sweden

Table S2. Characteristics of patients with a hematologic malignancy, population comparisons and sibling comparisons

	Patients n = 11,575			Population comparisons n = 57,727			Sibling comparisons n = 11,803		
**Follow-up time, years**	Median	Range	IQR	Median	Range	IQR	Median	Range	IQR
Leaving the parental home	5	0–36	4	5	0–36	3	5	0–36	3
Cohabitation with a non-marital partner	8	0–36	12	7	0–36	11	8	0–36	11
Cohabitation with a non-marital partner and parenting a child together	11	0–36	12	10	0–36	11	10	0–36	10
Marriage*	10	0–44	11	9	0–44	10	9	0–47	10
**Attained age at end of follow-up, years**									
Leaving the parental home	21	15–64	4	20	15–64	3	20	15–66	3
Cohabitation with a non-marital partner	25	16–64	12	24	16–64	12	25	16–69	11
Cohabitation with a non-marital partner and parenting a child together	28	16–64	13	27	16–64	11	27	16–69	11
Marriage*	27	16–63	10	26	16–64	10	26	16–67	9
**Age at first outcome event, years**									
Leaving the parental home	21	15–45	3	21	15–54	3	21	15–43	3
Cohabitation with a non-marital partner	23	17–53	5	22	16–56	5	22	16–54	5
Cohabitation with a non-marital partner and parenting a child together	28	17–50	7	28	16–55	7	27	16–50	6
Marriage*	28	16–58	7	28	16–60	7	28	16–58	7

* Registered partnerships and same sex marriages were incorporated into the same categories as married.

Table S3. Hazards ratios (HR) for outcomes leaving the parental home, cohabitation, and marriage with 95 % confidence intervals (CI) comparing patients with lymphoid leukemia with population comparisons.

Period of diagnosis	Leaving the parental home	Cohabitation with a non-marital partner	Cohabitation with a non-marital partner and parenting a child together	Marriage
	HR (95% CI)	HR (95% CI)	HR (95% CI)	HR (95% CI)
**< 1992**	0·81 (0·76–0·87)	0·72 (0·65–0·80)	0·67 (0·60–0·75)	0·71 (0·64–0·78)
**≥1992**	0·96 (0·90–1·02)	0·88 (0·78–0·99)	0·91 (0·78–1·07)	0·88 (0·74–1·04)
